# Customized First and Second Order Statistics Based Operators to Support Advanced Texture Analysis of MRI Images

**DOI:** 10.1155/2013/213901

**Published:** 2013-06-12

**Authors:** Danilo Avola, Luigi Cinque, Giuseppe Placidi

**Affiliations:** ^1^Department of Life, Health and Environmental Sciences, University of L'Aquila, Via Vetoio Coppito 2, 67100 L'Aquila, Italy; ^2^Department of Computer Science, Sapienza University of Rome, Via Salaria 113, 00198 Rome, Italy

## Abstract

Texture analysis is the process of highlighting key characteristics thus providing an exhaustive and unambiguous mathematical description of any object represented in a digital image. Each characteristic is connected to a specific property of the object. In some cases the mentioned properties represent aspects visually perceptible which can be detected by developing operators based on Computer Vision techniques. In other cases these properties are not visually perceptible and their computation is obtained by developing operators based on Image Understanding approaches. Pixels composing high quality medical images can be considered the result of a stochastic process since they represent morphological or physiological processes. Empirical observations have shown that these images have visually perceptible and hidden significant aspects. For these reasons, the operators can be developed by means of a statistical approach. In this paper we present a set of customized first and second order statistics based operators to perform advanced texture analysis of Magnetic Resonance Imaging (MRI) images. In particular, we specify the main rules defining the role of an operator and its relationship with other operators. Extensive experiments carried out on a wide dataset of MRI images of different body regions demonstrating usefulness and accuracy of the proposed approach are also reported.

## 1. Introduction

As it is well known, there is no univocal definition of texture [1–3]. This is due to the various and heterogeneous aspects involving the texture analysis process. In particular, two aspects influence more than other textural detection and recognition approaches: *image classification* and *target definition*. The first is used to classify images as belonging to the natural domain (e.g., cell movement) or artificial domain (e.g., gear movement) [4, 5]. Although this discrimination may seem obvious, it can present ambiguities due to both kind of image and acquisition method. The second aspect regards type and detail of the extracted key characteristics to define different textures. In some cases it could be sufficient to extract coarse information to highlight macroscopic textural aspects (i.e., macrotextures) of studied objects. In other cases it could be necessary to extract more detailed information to describe microscopic textural aspects (i.e., microtextures) [6–10]. In our context, we have analyzed MRI images of organs and tissues (e.g., brain). These images represent the morphological aspects of biological entities and they belong to the natural domain [11, 12]. In MRI, the pixels composing an image can be considered a reliable representation of water distribution in the body [13, 14], and the obtained images can be seen as the result of a stochastic process. This last assumption influences the choice of the reference model to be used for supporting the feature extraction method of the texture elements. The model is usually implemented by one of the following three descriptive approaches: *statistical*, *structural*, or *hybrid* [15–19]. It also includes the theoretical guidelines to develop the textural operators. The latter represents the core of the paper that will be detailed in the next section where the window based top-down image browsing method and the implementation of the customized first and second order statistics based operators will be described. 

The operators applied on a wide dataset containing *in vivo* MRI images were conceived to detect macroscopic and microscopic textural features supporting a complete and unambiguous mathematical description. Artifacts in the treated images could make the texture analysis process qualitatively ineffective since macro- and microvariations of the textural structures are linked to the spatial and contrast resolution of these images. Fortunately the current MRI scanners provide high quality images reasonably free from serious artifacts [20, 21]. 

Different works in the literature concerning the description of the natural domain by texture analysis processes [22–28], as well as our preliminary intuitions supported by some preparatory experimental observations on pixel arrangement of the MRI images, led us to focus on CV and IU based statistical operators. These included some operators to estimate the human visual perception of well-known textural features (e.g., *contrast*) and some operators to detect significant hidden textural features (e.g., *entropy*). Subsequently, our approach was refined by studying some remarkable works in texture analysis of MRI images. In a first set of works, detailed in [29–31], the authors described a method to model the objects contained in the layout of MRI images. After a training phase on a given image dataset to distinguish different target objects (e.g., brain mass, background), the approach was customized to achieve established tasks (e.g., layout segmentation) supporting a set of suitable Computer Aided Diagnosis (CAD) functionalities (e.g., mass identification). Their system architecture was composed of three main modules. The first (i.e., recognition) performed a feature extraction process on a set of template images to define numerical classes able to describe the different target objects composing the image layout. The second (i.e., classification) analyzed the source images, using the numerical classes defined in the previous module, to provide a classification of the different image zones. Finally, the last (i.e., segmentation) defined the boundaries between heterogeneous zones and merged homogeneous ones. Although their method included a set of statistical operators similar to those used in the present work, the authors did not produce any adequate explanation about operator potentiality, limits, and functional characteristics. Moreover, they neither showed any relationship between operators nor explained rules for their use. All these last aspects that make possible the reutilization of the operators to define new tasks on new target objects are addressed in the present work. Another reference work is [32], where the ability of the texture analysis in detecting micro- and macrovariations of the pixel distribution was described. The authors introduced an approach to classify multiple sclerosis lesions. Three imaging sequences were compared in quantitative analyses, including a comparison of anatomical levels of interest, variance between sequential slices, and two methods of region of interest drawing. They focused on the classification of white matter and multiple sclerosis lesions in determining the discriminatory power of textural parameters, thus providing high accuracy and reliable segmentation results. A work in the same direction is [33]: the concept, strategies, and considerations of MRI texture analysis were presented. The work summarized applications of texture analysis in multiple sclerosis as a measure of tissue integrity and its clinical relevance. The reported results showed that texture based approaches can be profitably used as tools of evaluating treatment benefits for patients suffering from this type of pathology. Another basic work showing the importance of the texture analysis applied on the brain is [34], where the authors focused their efforts on characterizing healthy and pathologic human brain tissues: white matter, gray matter, cerebrospinal fluid, tumors, and edema. In their approach each selected brain region of interest was characterized with both its mean gray level values and several texture parameters. Multivariate statistical analyses were then applied to discriminate each brain tissue type represented by its own set of texture parameters. Thanks to its rich morphological aspects, not only brain can be widely studied through texture analysis approaches but also other organs and tissues where they can appear less noticeable. In [35] the feasibility of texture analysis for the classification of liver cysts and hemangiomas on MRI images was shown. Texture features were derived by gray level histogram, cooccurrence and run-length matrix, gradient, autoregressive model, and wavelet transform obtaining results encouraging enough to plan further studies to investigate the value of texture based classification of other liver lesions (e.g., hepatocellular and cholangiocellular carcinoma). Another work following the same topic is [36], where a quantitative texture feature analysis of double contrast-enhanced MRI images to classify fibrosis was introduced. The approach, based on well-known analysis software (MaZda, [37]), was implemented to compute a large set of texture parameters for each image. A statistical regularization technique, generalized linear model path, was used to define an effective model based on texture features for dichotomous classification of fibrosis category. Different texture analysis approaches for liver segmentation and classification are reported in [38]. The work treated images coming from MRI as well as other imaging modalities (e.g., ultrasound) to support complete liver description to allow specific algorithms development to solve different diagnostic tasks. Studies similar to those carried out on brain and liver are increasingly performed on the heart to investigate its patterns and structures. For example, in [39] a method for automating the myocardial contours identification to optimize the detection and the tracking of the grid of tags within myocardium was presented. Endocardial and epicardial contours detection was based on the use of texture analysis and active contours models. In particular, the authors adopted the texture analysis to define energy maps supporting the whole segmentation process, and the results were very promising. A similar work was proposed in [40], where the authors described a dynamic texture based motion segmentation approach to address the challenging problem of heart localization and segmentation in 4D spatiotemporal cardiac images. The method introduced time-dependent dynamic constraints into model based segmentation, with the advantage of producing segmentation results both spatially and temporally consistent. Another interesting method focused on heart characteristics was described in [41], where an automatic segmentation of the left ventricle in 2D tagged MRI images based on contrast enhancement was presented. The method applied histogram modification and local contrast enhancement for improving contrast between tagged lines and nontagged tissue. The ventricular blood filled and tagged regions were isolated by subtracting gray minimum from maximum within a small window. In this context, wide feature values represented textured regions, and small values highlighted homogeneous ones, respectively. Finally, boundaries of the left ventricle were extracted. A last case study is focused on bone structures that more than others seem to present hidden and weak textural patterns. In [42] an approach to assess the ability of specific texture parameters to detect exercise load-associated differences in MRI images representing the neck cross-section was presented. In particular, the femoral neck trabecular bone at the level of the insertion of articular capsule was divided manually into regions of interest representing four anatomical sectors: anterior, posterior, superior, and inferior. Selected cooccurrence matrix based texture parameters were used to evaluate differences in apparent trabecular structure between the exercise loading groups and anatomical sectors of the femoral neck. The reported results showed qualitative and quantitative evaluations in detecting and classifying structural differences in trabecular bone associated with specific exercise loading. Another remarkable work was [43], where the authors described a unified framework for automatic segmentation of intervertebral disks of scoliotic spines from different types of magnetic resonance image sequences. Their method exploited a combination of statistical and spectral texture features to discriminate closed regions representing intervertebral disks from background in acquired images of the spine. A set of texture features were extracted from every closed region obtained from an automatic segmentation procedure based on the watershed approach. The authors validated their approach by using a supervised *k*-nearest-neighbor classifier on a wide number of images. A final work on this topic was [44], where a method for automatic segmentation of the tibia and femur in clinical magnetic resonance images of knees was presented. The texture information was incorporated into an active contours framework through the use of vector-valued geodesic snakes with local variance as a second value at each pixel, in addition to intensity. The use of this additional information allowed to develop a system to better handle noise and nonuniform intensities found within the structures to be segmented. Besides those mentioned, other works [45–49] were focused on the texture analysis on medical images to accomplish heterogeneous tasks; among these we considered only approaches related to four specific anatomical regions (*brain*, *heart*, *liver*, and *bones*) since they represented the images on which we used the customized first and second order statistics based operators. Unlike the works cited above that adopted texture analysis to achieve specific targets (e.g., lesion detection, mass classification, and 3D reconstruction), our intent was to provide a set of customized first and second order statistics based operators to support the definition of any new task. We designed a general purpose texture analysis approach to evaluate the behavior of each operator on the established medical domains. In addition, having each operator a different numerical feedback when applied on images belonging to a specific domain, we defined some main rules specifying the role of an operator and its relationship with others in distinguishing different textural aspects. For all these reasons, our approach is not directly comparable with others since any work regarding roles and rules of operators is given. Moreover, our method to extract textural features can be considered novel with respect to the current state of the art.

The paper is structured as follows.  Section 2 details the proposed texture analysis process, including the first and second order statistics based operators. Information about their potentiality, limits, and functional characteristics are also reported.  Section 3 summarizes and discusses the extensive experimental results showing the application of each operator on MRI images of brain, heart, liver, and bones. Finally, Section 4 concludes the paper.

## 2. Materials and Methods

This section details the designed texture analysis process. The mentioned process is the same on each established type of image (i.e., brain, heart, liver, and bones), where only the definition of some parameters has to be adjusted depending on both the specific type of image (e.g., brain or heart) and the fixed targets (e.g., brain mass or heart lesions identification). Being different targets heterogeneous and hugely numerous, our purpose is to provide a guideline on *why* and *how* to adopt the developed method and related operators without a specific case study. In the rest of the paper, we suppose that every image (e.g., brain) is analyzed by our approach in a supervised way in order to study the numerical feedback of each operator according to different constitutive parts of the image (e.g., background, cerebral tissue, on skull). This step is fundamental since it leads the segmentation activity, the basic process to support each complex task in biomedical image analysis [50, 51].

Each image is entirely browsed by a window (recognition window, RW) of fixed size (i.e., *n*_*a*_ × *n*_*b*_, *n*_*a*_, *n*_*b*_ ∈ *N*), in top-to-down and left-to-right way without overlapping. It is important to note that any other non-overlapping browser strategy would give the same results. The size of the window can change depending on the specific target, the type of the image, and its spatial resolution. The first aspect points out that the patterns associated to different textural analysis processes (e.g., ventricular lesion discovery, semilunar valve identification) on a given dataset of images (e.g., heart) can be detected by a suitable set of operators trained to identify different pixel configurations. The second highlights that the study of the micro- and macrotextural components depends on the specific organ or tissue. Finally, the last aspect draws attention to the relationship between the scanning window and the image spatial resolution. Our approach is conceived to face the first two aspects, while in the experimental section (Section 3) we show the image technical characteristics and the related assumptions for the browsing window size. 

Each RW contains a set of pixels (i.e., *p*_*i*_) elaborated from the whole set of functions (i.e., operators: *f*_*i*_) to provide a corresponding set of numerical values (i.e., *Q*_*j*, *f*_*i*__), each one representing a characterization of the mentioned pixels depending on the adopted operator (see the next subsection). Formally, we can summarize the general operation of the developed functions as follows:

(1) $$\begin{array}{c} Q_{{j,f}_{i}}=f_{i}\left(p_{0},p_{1},p_{2},\ldots ,p_{n}\right), \end{array}$$

where *Q*_*j*, *f*_*i*__ ∈ *N* ^3^ represents the new pixel *j* ∈ *N* of the new image (i.e., feature map, FM_*f*_*i*__) depending on the function *f*_*i*_. The pixel is structured in (*x*, *y*, *z*) ∈ *N* ^3^ where (*x*, *y*) ∈ *N* ^2^ sets the spatial position of the pixel, while *z* ∈ *N* sets the amplitude value. *f*_*i*_ : *N* ^*n*^ × *N* represents the operator *i* ∈ *N* with domain in *N* ^*n*^ and codomain *N*, and *n* ∈ *N* represents the number of elaborated pixels (i.e., pixels contained in RW). *p*_*i*_ ∈ *N* ^3^ represents a pixel contained in the RW having the same structure of *Q*_*j*, *f*_*i*__.

**Figure 1:**
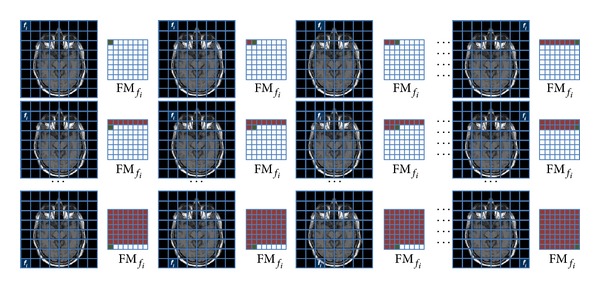
The source image is browsed by the operator *f*_*i*_ to obtain the related feature map FM_*f*_*i*__. The map is subsampled as each RW provides a pixel as result. In the example the source image is analyzed by 64 RW thus providing a subsampled 8 × 8 image.

As shown in Figure 1, the elaboration of each source image provides a subsampled FM_*f*_*i*__. The numerical values contained in this new map represent the behavior of the operator *f*_*i*_ in processing the different pixel configurations belonging to the different constitutive parts of the image. By performing this operation on the whole dataset of images, we obtain an equal number of subsampled images (i.e., feature space, FS_*f*_*i*__) related to the operator *f*_*i*_; furthermore, following the same method, we can obtain the feature space related to each specific operator (FS_*f*_1__, FS_*f*_2__,…, FS_*f*_*m*__, *m* ∈ *N*). These spaces suitably managed and interpreted represent the basic statistical information to implement an ad hoc mathematical model to achieve different targets (e.g., brain mass identification). We adopt the pyramid based texture analysis approach [52, 53] (i.e., subsampling strategy) to avoid redundant values in modeling definition. Despite this, the proposed approach is fully parametric thus allowing RW shape and size modification, image browsing process setting (i.e., with and without overlap), and number of the pyramidal levels choice. In Section 3 we detail and fix the whole set of parameters.

### 2.1. Operator Implementation

This section shows the CV and IU textural operators customized after our investigative experience regarding the established domains: brain, heart, liver, and bones. Since the browsing process determines the (*x*, *y*) ∈ *N* ^2^ position of the new pixel within the related feature map, in the following formalization we can omit it. Finally, we highlight that the observations related to the numerical feedback of the operators have to be considered tied to the established natural domains without general implications on different kinds of images.

The first two operators we consider are based on the first order statistic, specifically, *N-order moment* (*M*_*n*_1__) and *N-order central moment* (*C*_*n*_2__):

(2) $$\begin{array}{c} M_{\left(n_{1}\right)}=\sum_{i\;=\;0}^{L-1}i^{n_{1}}\cdot p\left(i\right),\;\;C_{\left(n_{2}\right)}=\sum_{i\;=\;0}^{L-1}{\left(i-M_{n_{1}}\right)}^{n_{2}}\cdot p\left(i\right), \end{array}$$

where *p*(*i*) represents the probability that the gray level *i* ∈ [0 ⋯ *L* − 1] appears within the RW, *L* represents the number of levels of color in the source image, and *n*_1_, *n*_2_ represent the orders of the *M*_(*n*_1_)_ and *C*_(*n*_2_)_, respectively.

The following constraints must hold:

(3) $$\begin{array}{c} \forall i\in \left[0\cdots L-1\right]\subset N,\;0\leq p\left(i\right)\leq 1,\\ \sum_{i\;=\;0}^{L-1}p\left(i\right)=1;\;n_{1},n_{2}\in N. \end{array}$$

Actually, they are not properly textural operators, since their task is only to measure the informative content of different image zones. In particular, the first operator (*M*_(*n*_1_)_) calculates the average of the levels of color related to the pixels contained within the RW; the second operator (*C*_(*n*_2_)_) measures the amplitude dispersion that the pixels contained within the RW have compared to their average (i.e., *M*_(*n*_1_)_). The order of the two operators leads the dynamic and the detail of the obtained numerical values. In our context, these operators are mainly used to distinguish the background of an image from the rest of image. In fact, the background image zones are usually characterized by very low *M*_(*n*_1_)_ values and low *C*_(*n*_2_)_ values. Moreover, these operators are also used to support the discrimination of regions of interest (ROIs) which present high *M*_(*n*_1_)_ and very high *C*_(*n*_2_)_. A last use of these operators regards the detection of zones belonging to ROIs and not to background; this generally occurs in those areas across two different textural zones (e.g., cerebral tissue and skull) which present low *M*_(*n*_1_)_ and high *C*_(*n*_2_)_.

**Figure 2:**
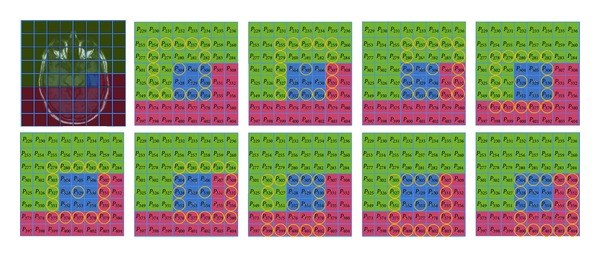
Variation of the Haralick et al. approach which considers all the possible directions and not only the cardinal ones. In the example the RW contains 9 pixels; each circumference provides 16 pair of pixels; therefore the current RW provides 144 values to the co-occurrence matrix.

The rest of the operators introduced in this section are fully texture based since they work both on spatial disposition and amplitude value of the pixels contained within the RW. They are based on the Haralick et al. studies [54, 55] which were oriented to discriminate different meaningful textural features through the utilization of co-occurrence matrices. Currently, there are more advanced methods to discover textural features on natural images [56, 57]; despite this, our preliminary studies on the established domains have led us in using the proposed approach which seems completely suitable and profitable. Experimental results supported our efforts. Actually, we have adopted a variation of the classical approach which is designed to detect textural features by considering only four fixed directions (i.e., 0°, 45°, 90°, and 135°). As shown in Figure 2, our method considers each pixel contained within the RW as the center of a discrete circumference whose radius (*d*) can be freely defined (in Figure 2: *d* = 2). Each pair of points formed by the current central point and the one located on the perimeter of the circumference will increase the related position within the cooccurrence matrix. A careful analysis of the numerical results obtained by this approach allows for obtaining both textural aspects related to the pixels contained within the RW and contextual information of the pixels positioned around the RW. In this way, changing the parameter *d* of the RW, we can analyze the dynamic of the textural variations in a significant neighborhood of the RW.

The first two textural operators belonging to the second order statistic are customized to emulate two main visual perceptions related to the CV field [58], specifically, *homogeneity * (HG(*d*)_(*n*_3_)_) and *contrast* (CT(*d*)_(*n*_4_,*n*_5_)_):

(4) $$\begin{array}{c} {\text{HG}\left(d\right)}_{\left(n_{3}\right)}=\sum_{i\;=\;0}^{L-1}\;\sum_{j\;=\;0}^{L-1}{\left[p_{d}\left(i,j\right)\right]}^{n_{3}},\\ {\text{CT}\left(d\right)}_{\left(n_{4},n_{5}\right)}=\sum_{i\;=\;0}^{L-1}\;\sum_{j\;=\;0}^{L-1}{\left|i-j\right|}^{n_{4}}\cdot {\left[p_{d}\left(i,j\right)\right]}^{n_{5}}\;, \end{array}$$

where *p*_*d*_(*i*, *j*) represents the probability that two pixels, with distance *d*, have, respectively, *i* ∈ [0 ⋯ *L* − 1] and *j* ∈ [0 ⋯ *L* − 1] amplitude values. *n*_3_, *n*_4_, and *n*_5_ represent the parameters of the generalized HG(*d*)_(*n*_3_)_ and CT(*d*)_(*n*_4_,*n*_5_)_.

The following constraints must hold:

(5) $$\begin{array}{c} \forall \left(i,j\right)\in \left[0\cdots L-1\right]\times \left[0\cdots L-1\right]\subset N^{2},\;0\leq p_{d}\left(i,j\right)\leq 1,\\ \sum_{i\;=\;0}^{L-1}p_{d}\left(i,j\right)=1,\;n_{3},n_{4},n_{5}\in N. \end{array}$$

HG(*d*)_(*n*_3_)_ measures the degree of uniformity associated to the different image zones. It provides high values on those zones having a high homogeneity level, while low values denote zones highly disconnected, as well as zones containing different textures. By comparing numerical values of adjacent image zones, the operator can measure the changing of textural structures covering a portion of an image. In particular high or low variations of the analyzed image zones reflect light or wide changes in textural structures, respectively. The results obtained from the described operator are highly dependent on the setting of the radius *d* since in natural domains, including the established ones, the uniformity is a characteristic with rapid changes depending on the local pixel distribution. Finally, the parameter *n*_3_ serves to define the dynamic of the obtained results: it has to be customized according to both image kind and specific target.

CT(*d*)_(*n*_4_,*n*_5_)_ measures the amplitude variation between different image zones. When applied to textures composed of pixels with constant intensities (i.e., similar amplitude values), it provides very low values (CT(*d*)_(*n*_4_,*n*_5_)_ ≈ 0). On the contrary, the operator provides high numerical values on image zones having high variations of the pixel intensities, thus reflecting amplitude changes and density transitions. From a different point of view, this operator can be seen as a measure of how textural components are structured on different image zones. In fact, the passage between very high and very low values belonging to different zones reflects a definite structured pattern while constant values do not show any significant modification. Also in this case, the value of the radius *d* influences the result of the operator. This is due to the possible amplitude variations of the texture. However, the operator shows significant diversifications only considering distant radius values. Finally, the parameters *n*_4_ and *n*_5_ have to be experimentally investigated to define the sensibility of the operator in discovering the structural variations of the involved patterns.

In our context, HG(*d*)_(*n*_3_)_ and CT(*d*)_(*n*_4_,*n*_5_)_ can be seen as the measure of macrotextural aspects of the MRI images tied to the visual perception to immediately identify the basic components contained within the layout of the treated images.

The other two textural operators belonging to the second order statistics are customized to determine two hidden significant features useful to identify both the period and the size of the involved patterns, specifically, *inverse difference* (ID(*d*)_(*n*_6_,*n*_7_)_) and *entropy* (ET(*d*)_(*n*_8_,*n*_9_)_):

(6) $$\begin{array}{c} {\text{ID}\left(d\right)}_{\left(n_{6},n_{7}\right)}=\sum_{i\;=\;0}^{L-1}\;\sum_{j\;=\;0}^{L-1}\frac{{\left[p_{d}\left(i,j\right)\right]}^{n_{6}}}{{1+\left(i-j\right)}^{n_{7}}},\\ {\text{ET}\left(d\right)}_{\left(n_{8},n_{9}\right)}=-\sum_{i\;=\;0}^{L-1}\;\sum_{j\;=\;0}^{L-1}{\left[p_{d}\left(i,j\right)\right]}^{n_{8}}\cdot {\left[\log_{k_{1}}\left(p_{d}\left(i,j\right)\right)\right]}^{n_{9}}\;, \end{array}$$

where *n*_6_, *n*_7_, *n*_8_, *n*_9_, *k*_1_ ∈ *N* represent the parameters of the generalized ID(*d*)_(*n*_6_,*n*_7_)_ and ET(*d*)_(*n*_8_,*n*_9_)_. The other terms are defined as in (5).

ID(*d*)_(*n*_6_,*n*_7_)_ measures and characterizes the local distribution of pixels within an image zone. The set of numerical values it provides can be used to define a specific pixel configuration and its repetitions. In this operator the parameters *n*_6_, *n*_7_, and *d* are adopted to fix experimentally one or more patterns within the source image by considering their scale, sizes, and rotations. This operator is conceived to catch high level of details. For this reason it is suitable to define microtextural aspects of the analyzed zones.

ET(*d*)_(*n*_8_,*n*_9_)_ measures the degree of disorder related to different image zones. Its values are directly proportional to the randomness level detected within the analyzed zones. Also in this case the parameters *n*_8_, *n*_9_, *k*_1_, and *d* have to be empirically fixed to support the pattern definition. In particular, the following strategy provided profitable results to support the identification of the pixel configuration: starting from image zones having high homogeneity levels (HG(*d*)_(*n*_3_)_), the behavior of this operator can be analyzed according to the progressive increments of *d*; in this way we can provide the structural changes of the distribution composing the identified patterns.

In our context, ID(*d*)_(*n*_6_,*n*_7_)_ and ET(*d*)_(*n*_8_,*n*_9_)_ are used to define the basic features of the microtextural aspects of the image zones, in particular, to distinguish different significant textural structures composing the given images (according to the fixed tasks).

Empirical experiences have allowed us to consider two other textural operators to support the working of the previous ones. These operators do not have a specific meaning: they are utilized to increase the detail and the reliability of the proposed second order statistics based operators, specifically, *correlation* (CR(*d*)_(*n*_10_,*n*_11_)_) and *difference entropy* (DE(*d*)_(*n*_12_,*n*_13_)_):

(7) $$\begin{array}{c} {\text{CR}\left(d\right)}_{\left(n_{10},n_{11}\right)}=\sum_{i\;=\;0}^{L-1}\;\sum_{j\;=\;0}^{L-1}\frac{\left(i-\mu_{x}\right)\cdot \left(i-\mu_{y}\right)\cdot {\left[p_{d}\left(i,j\right)\right]}^{n_{10}}}{{\left[\sigma_{x}\cdot \sigma_{y}\right]}^{n_{11}}},\\ {\text{DE}\left(d\right)}_{\left(n_{12},n_{13}\right)}=-\sum_{i\;=\;0}^{L-1}{\left[p_{x-y}\left(i\right)\right]}^{n_{12}}\cdot {\left[\log_{k_{2}}\left(p_{x-y}\left(i\right)\right)\right]}^{n_{13}}, \end{array}$$

where *n*_10_, *n*_11_, *n*_12_, *n*_13_, *k*_2_ ∈ *N* represent the parameters of the generalized CR(*d*)_(*n*_10_,*n*_11_)_ and DE(*d*)_(*n*_12_,*n*_13_)_. The other terms are defined as in (5). Moreover

(8) $$\begin{array}{c} \mu_{x}=\sum_{i\;=\;0}^{L-1}\;\sum_{j\;=\;0}^{L-1}\left[i\cdot \left(p_{d}\left(i,j\right)\right)\right],\\ \sigma_{x}=\sqrt{\sum_{i\;=\;0}^{L-1}\sum_{j\;=\;0}^{L-1}\left[{\left(i-\mu_{x}\right)}^{2}\cdot \left(p_{d}\left(i,j\right)\right)\right]},\\ \mu_{y}=\sum_{i\;=\;0}^{L-1}\;\sum_{j\;=\;0}^{L-1}\left[j\cdot \left(p_{d}\left(i,j\right)\right)\right],\\ \sigma_{y}=\sqrt{\sum_{i\;=\;0}^{L-1}\sum_{j\;=\;0}^{L-1}\left[{\left(j-\mu_{y}\right)}^{2}\cdot \left(p_{d}\left(i,j\right)\right)\right]},\\ p_{x-y}\left(k\right)=\sum_{i\;=\;0}^{L}\;\sum_{j\;=\;0}^{L}{\left[p_{d}\left(i,j\right)\right]}^{q},\;\text{where}\;\;\left|i-j\right|=k. \end{array}$$

CR(*d*)_(*n*_10_,*n*_11_)_ and DE(*d*)_(*n*_12_,*n*_13_)_ are mainly used to recognize relationships between near RWs and different discovered patterns, respectively. In other words, the first can be used to identify spatial constraints of RWs composing the same pattern, while the second can be adopted to determine the spatial constraints of different textural patterns.

In this context, it is important to note that the whole set of operators have different degrees of dependence. This means that the results of each operator have to be considered jointly with those provided from the others. A single operator is only able to describe the general features of a complex texture. To identify more patterns, different textural aspects have to be adopted within the same mathematical model.  Table 1 shows the dependence between operators which can be considered as a general guideline to adopt the customized set of textural operators to implement specific tasks related to the established domains. With reference to the first row of Table 1, we can observe that the operator *M*_(*n*_1_)_ is strongly joint to the operator *C*_(*n*_2_)_; this means that they have to be jointly evaluated to provide a reliable numerical result. Subsequently, the other dependent levels highlight the order by which the values coming from *M*_(*n*_1_)_ have to be compared with those provided from the other operators. This approach is aimed at refining the obtained results. When two or more operators have similar dependent level, the choice is empirically performed according to the specific task. The other rows can be interpreted in the same way. Finally, we highlight that the results summarized in Table 1 are derived by analyzing the behavior of the operators on the established domains according to the segmentation task. For this reason, they can be adopted as general guidelines on each defined new task.

## 3. Results and Discussion

In order to define qualitative and technical aspects of the proposed approach, experimental results were obtained from a wide dataset containing images of brain, heart, liver, and bones. In particular, the experimental phase was divided into three sessions: basic parameter definition, model parameter definition, and qualitative response. The first served to identify the basic parameters through which the source images had to be browsed; the second focused on the parameter definition of each first and second order statistics based operator according to a specific natural domain; finally, the third focused on the qualitative aspects of the approach with respect to a specific basic task (i.e., segmentation). All the experimental sessions were performed using MRI transversal *T*_1_ weighted, *T*_2_ weighted, and proton density (PD) images having 8 bit (i.e., *L* = 256) and 512 × 512 pixels.

### 3.1. Basic Parameter Definition

Table 2 summarizes the preliminary analysis which supported the definition of several critical parameters of the proposed approach. We used 110 images, collected by 41 different patients suitably subdivided within the four natural domains. As previously mentioned, we have focused on the segmentation task since it represents the basic step of the image processing. Dimension and shape of the RW represent crucial aspects. An RW too wide causes the loss of textural details; an RW too small is unable to detect textural features. On each image both variance (i.e., *C*_2_) and basic entropy (i.e., ET(2)_(2, 3)_) values of the pixel distribution were empirically evaluated with the aim of maximizing them and at the same time minimizing the RW. These observations have shown that a square RW sized 6 × 6 pixels can provide the best solution to catch the micro- and macrotextural aspects from all studied domains. In some cases, when noise and low information occur within specific image zones, it can be useful to enlarge or decrease (of one unit) the RW size. Moreover, these observations have also highlighted that the image browsing can be performed in conventional mode (i.e., top-to-down and left-to-right) without overlapping, while maintaining the integrity of the extracted textural information content. Only in some cases, regarding bones (in particular, the hand) it can be necessary to browse the image with an overlapping strategy to infer information about structures and patterns. For the same reasons, overlapping strategy can be applied on images having very low textural variations. In this experimental session we obtained both base (*L*_0_) and first (*L*_1_) pyramidal levels. The base level was considered to analyze variance and entropy values, while the first level was exploited to support the definition of image zones having low information content. Finally, we have also deduced that the usefulness of the first level is directly proportional to dynamical aspects of the involved textures. In fact, in brain and heart images which have high variation of the textural structures, the first level of the pyramid can provide distinguishing information than liver and bone images which have textural structures less complex and heterogeneous.

### 3.2. Model Parameter Definition

Table 3 summarizes the parametric definition of the first and second statistics based operators which define our statistical model. We used 380 images, collected by 160 different patients. Also in this case, the images were suitably subdivided within the four natural domains. In this session we analyzed different patients (and related images) from the previous ones with the aim of obtaining a more objective investigation. Moreover, this session was implemented by developing an ad hoc machine-learning supervised algorithm [59] able to highlight the differences of the numerical feedbacks provided by the set of operators during the analysis process. Initially, each parameter of each operator was fixed to 1 in order to obtain an initial state of the algorithm. Subsequently, a skilled user modified variance (i.e., *C*_2_) and basic entropy (i.e., ET(2)_(2, 3)_) values to identify the different image zones having the highest numerical feedbacks. Starting from these values the skilled user implemented a feature vector composed of all operators. The parameters contained within the vector were methodically increased or decreased to produce different feature spaces useful to perform the current task (in this case, segmentation). In other words, the skilled user modified, in a supervised way, the parameters contained within the vector taking into account the role and dependence table (Table 1) of each operator. The main aim of the parameter variation was to increase the numerical feedback of the textural operators decreasing the variation among “independent” (or less dependent) operators. Once several feature spaces were computed (i.e., states of the algorithm), the approach provided a final set of numerical intervals. These intervals can be considered as the mathematical model describing the fixed task. In particular, each object contained within the layout of an image can be defined by a specific set of numerical values obtained by considering the whole set of operators and related variations due to the different parameters. Despite the large amount of parameters shown in Table 3, just a minor part of them with a restricted set of values define the main features of the objects. For example, the discrimination of the background from the informative content is almost always performed by using *M*_(*n*_1_)_ and *C*_(*n*_2_)_ with *n*_1_ = 1 and *n*_2_ = 2, respectively. The other values are used to solve particular cases due to noise or artifacts. Similarly, the radius adopted to the different operators to describe the related textural features is almost always fixed on *d* = 2 and *d* = 3; the other values are used to solve particular issues tied to the boundary detection of two different textural zones. However, the whole set of parameters shown in the Table 3 can be considered as a guideline to implement and customize multipurpose operators. Finally, we observe that when a skilled user defines a new task (e.g., mass detection) on a well-established domain, (e.g., brain) a new training stage has to be performed to define the new reference mathematical model. 

### 3.3. Qualitative Response

**Figure 3:**
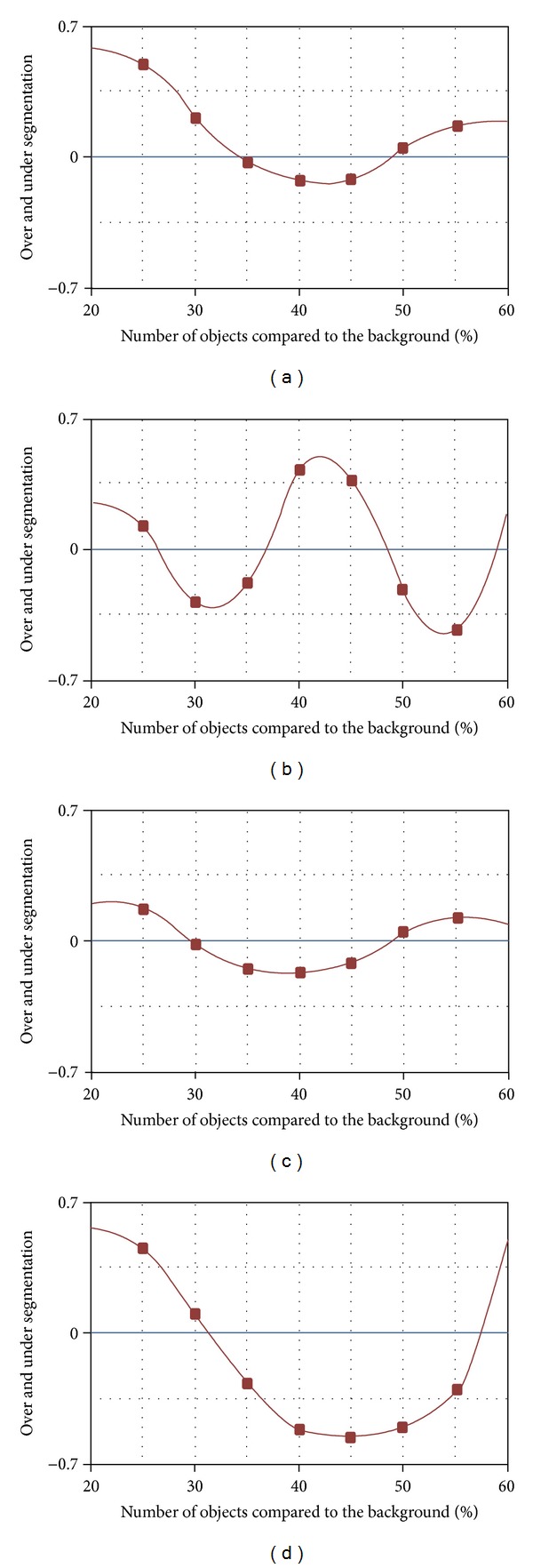
Qualitative response on (a) brain, (b) heart, (c) liver, and (d) bone.

Figure 3 summarizes the qualitative response of the proposed approach in relation to the assigned basic task: the segmentation. In order to obtain comparable results independently of the specific natural domain, the set of images was chosen according to some simple morphological rules: (a) high stationarity of the texture, (b) substantial availability of the target objects, and (c) avoiding ambiguities related to the transition of different objects. All these aspects can be reasonably satisfied considering images belonging to “middle” transversal scanner planes where organs and tissues represent a wide portion of the whole image (not less than 20%). We report four graphics (Figure 3) representing the qualitative ratio between the pixels belonging to the real objects and the ones belonging to the segmented objects. In particular, Figures 3(a), 3(b), 3(c), and 3(d) represent the qualitative measurements of the brain, heart, liver, and bones, respectively. Note that while the abscissa in the first three cases represents the increasing amount of objects due to the transversal scanning plan, in the last case it represents the amount of objects due to a supervised choice of the images. The four figures show that the segmentation error (over and/or under segmentation) is less than 8% on the whole set of images by using the same set of operators.

## 4. Conclusion

Texture analysis of MRI images supports their exhaustive and unambiguous mathematical description. The base of this process is composed of a set of feature extractors to detect the key characteristics related to the objects contained within the image layout. These characteristics change depending on the established task (e.g., volume evaluation, lesions identification); despite this, our parametric approach designed for specific MRI images (i.e., brain, heart, liver, and bones) and the developed set of customizable textural operators can jointly provide a numerical interpretation of the images according to the specific task. This numerical interpretation represents a tool to describe different models to implement heterogeneous CAD functionalities (e.g., mass identification). To prove the usefulness and the accuracy of the proposed approach, we have fixed and tested the segmentation task on the analyzed domains; these experimental sessions allowed providing a set of information (i.e., roles, rules, and dependences) on the developed operators which can be used as guidelines to implement new tasks and CAD functionalities.

## Figures and Tables

**Table 1:** Relationship between the customized first and second order statistics based operators. The value from 1 (low) to 4 (high) points out the dependence level between two operators.

Operators	*M*_(*n*_1_)_	*C*_(*n*_2_)_	HG(*d*)_(*n*_3_)_	CT(*d*)_(*n*_4,_*n*_5_)_	ID(*d*)_(*n*_6_,*n*_7_)_	ET(*d*)_(*n*_8,_*n*_9_)_	CR(*d*)_(*n*_10_,*n*_11_)_	DE(*d*)_(*n*_12_,*n*_13_)_
*M*_(*n*_1_)_	⋯	4	3	3	2	2	1	2
*C*_(*n*_2_)_	4	⋯	2	3	2	3	2	2
HG(*d*)_(*n*_3_)_	3	2	⋯	4	3	3	1	1
CT(*d*)_(*n*_4,_*n*_5_)_	3	3	4	⋯	3	2	2	2
ID(*d*)_(*n*_6_,*n*_7_)_	2	2	3	3	⋯	4	3	2
ET(*d*)_(*n*_8,_*n*_9_)_	2	3	3	2	4	⋯	3	3
CR(*d*)_(*n*_10_,*n*_11_)_	1	2	1	2	3	3	⋯	4
DE(*d*)_(*n*_12_,*n*_13_)_	2	2	1	2	2	3	4	⋯

**Table 2:** I° experimental session: basic parameter definition.

Basic parameter definition									
Body regions	Training patients	Training images	Task	Recognition window (RW)		Image scanning process		Pyramid level	
				Shape	Size	Mode	Type	Levels	*L*_1_ usefulness
Brain	15	35	Segmentation	Square	*n*_*a*_ × *n*_*b*_=6×6	Top-to-down Left-to-right	Without overlapping	*L*_0_ and *L*_1_	Very high
Heart	10	30	Segmentation	Square	*n*_*a*_ × *n*_*b*_=6×6 *n*_*a*_ × *n*_*b*_=7×7	Top-to-down Left-to-right	Without overlapping	*L*_0_ and *L*_1_	High
Liver	8	25	Segmentation	Square	*n*_*a*_ × *n*_*b*_=6×6 *n*_*a*_ × *n*_*b*_=5×5	Top-to-down Left-to-right	Without overlapping	*L*_0_ and *L*_1_	Middle
Bone	8	20	Segmentation	Square	*n*_*a*_ × *n*_*b*_=6×6 *n*_*a*_ × *n*_*b*_=5×5	Top-to-down Left-to-right	With and without overlapping	*L*_0_ and *L*_1_	Low

**Table 3:** II° experimental session: model parameter definition.

Model parameter definition										
Body regions	Training patients	Training images	First and second order statistics based operators							
			*M*_(*n*_1_)_	*C*_(*n*_2_)_	HG(*d*)_(*n*_3_)_	CT(*d*)_(*n*_4,_*n*_5_)_	ID(*d*)_(*n*_6_,*n*_7_)_	ET(*d*)_(*n*_8,_*n*_9_)_	CR(*d*)_(*n*_10_,*n*_11_ )_	DE(*d*)_(*n*_12_,*n*_13_ )_
Brain	55	135	*n*_1_ = 1,2	*n*_2_ = 2,3	*n*_3_ = 2,3 *d* = 1,2, 3,4	*n*_4_ = 2,3 *n*_5_ = 1,2 *d* = 1,2, 3,4	*n*_6_ = 1,2, 3 *n*_7_ = 2 *d* = 1,2, 3,4	*n*_8_ = 1,2 *n*_9_ = 1,2 *k*_1_ = 2 *d* = 1,2, 3,4	*n*_10_ = 1,2, 3 *n*_11_ = 1,3 *d* = 1,3	*n*_12_ = 2,3, 4 *n*_13_ = 2,4 *k*_2_ = 2 *d* = 2,4

Heart	45	105	*n*_1_ = 1,2	*n*_2_ = 2,3	*n*_3_ = 2,3 *d* = 1,2, 3,4	*n*_4_ = 2,3 *n*_5_ = 1,2 *d* = 1,2, 3,4	*n*_6_ = 2,3 *n*_7_ = 2 *d* = 2,4	*n*_8_ = 2,3 *n*_9_ = 2 *k*_1_ = 2 *d* = 2,4	*n*_10_ = 1,3 *n*_11_ = 2,3 *d* = 1,2, 4	*n*_12_ = 1,4 *n*_13_ = 2,4 *k*_2_ = 2 *d* = 1,2, 4

Liver	35	85	*n*_1_ = 1,2	*n*_2_ = 2,3	*n*_3_ = 2,3, 4 *d* = 1,2, 3,4	*n*_4_ = 2,3 *n*_5_ = 1,2 *d* = 1,2, 3,4	*n*_6_ = 1,3 *n*_7_ = 3 *d* = 1,2, 3,4	*n*_8_ = 1,2, 3 *n*_9_ = 3 *k*_1_ = 2 *d* = 2,3, 4	*n*_10_ = 2,4 *n*_11_ = 3 *d* = 1,2, 4,6	*n*_12_ = 1,4 *n*_13_ = 2 *k*_2_ = 2 *d* = 1,2, 3,4

Bone	25	55	*n*_1_ = 1,2	*n*_2_ = 2,3, 4	*n*_3_ = 2,3, 4 *d* = 2,4, 6	*n*_4_ = 2,3 *n*_5_ = 2 *d* = 2,3, 4	*n*_6_ = 1,3 *n*_7_ = 2 *d* = 2,3, 4	*n*_8_ = 2,3, 4 *n*_9_ = 3 *k*_1_ = 2 *d* = 2,4	*n*_10_ = 2,4 *n*_11_ = 2,4, 6 *d* = 2,4	*n*_12_ = 3 *n*_13_ = 3 *k*_2_ = 2 *d* = 2,4
